# Notes on Medical Matters and Medical Men in Paris

**Published:** 1848-03

**Authors:** David W. Yandell

**Affiliations:** Louisville, Kentucky; Paris


**Art. III.**—*Notes on Medical Matters and Medical Men in Paris.* By David W. Yandell, M.D., of Louisville, Ky.

*On Induced Abortion in cases of Contracted Pelvis. From the Clinique of Professor Paul Dubois.*—Among the various means which the obstetric art opposes to the dangers incident to contracted pelvis, there is one which consists in the induction of the premature expulsion of the fœtus.

In most cases this expulsion is solicited at a period of pregnancy sufficiently advanced, that, the fœtus being viable, the operation may not prove unavoidably prejudicial to the preservation of its existence; the process thus effected is called *artificial premature labor.*

In some cases, however, it has been thought necessary to bring on this expulsion during the very first periods of pregnancy, when the fœtus has not yet acquired the necessary development for the performance (if it may be so termed) of extra-uterine life; that, is during the first seven months, the operation being then named *induced abortion.*

*Artificial premature labor* is, in general, only resorted to in cases of moderate contraction of the pelvis, and has for its end the preservation of the life both of the mother and child. It was long before this process, thus understood, was admitted into science—but at this day, its usefulness and justifiableness can no longer be the subject of serious contention.

The subject of this memoir, *induced abortion*, is on the contrary, only applicable to cases where the contraction of the pelvis is extremely marked; its inevitable result is the sacrifice of an undeveloped ovum, either in the embryotic or fœtal stage; but it is resorted to with the hope of saving the mother. It will be easily understood, from what has just been said, that this operation would not be quietly admitted into practice; that it met with very decided opposition; that many most serious objections were urged against it, and that its very profound abuse intimidated the moral sense of many accoucheurs. Indeed, the question we now propose investigating, is from its very nature one of the most serious and delicate topics of discussion that can be found in the whole science of obstetrics.

The first advice for bringing on abortion with the view of avoiding certain dangers of full-term parturition, is now very ancient. The following prescriptions from Aspasia, a celebrated Greek courtesan, as quoted by Ætius, may be found applicable to our subject, so far, at least, as they were intended to prevent the perilous consequences arising from obstacles esteemed insurmountable by the natural efforts during labor. We find in Ætius the following:

Quandam mulieres et amsi concipiant, in partu tamen periclitantur, sive ab uteri parvitaten ut *ab id* factam perficere non possit, sive ab colli ejusdem angustiam, sive quod tuberculum aut tale quiddam in ejusdem ostio exortum est quod partum impedit; atque hoc sane optimi fecerint si a partu omnius caverint. At si conciperint satius et factum corrumpere quam excidere.

And a little further on—

Si mulier at gignendum factum nepta per negligentiam conciperit, vehementissmis motibus uti jubeatur *et ut saliat* ac *gravissima onera levet* et *ut decoctionibus urinam* ac *menses prælectantibus* atque *alvum sub ducentibus assidue utatur* . . . quod si hoc nihil proficerit ad *validiora auxilia* pergendum erit, etc., etc.

I have only given the smallest part of the numerous means prescribed by Aspasia for inducing abortion in the earlier periods of pregnancy; the list is very long and exceedingly curious, and it would appear that the knowledge of this matron on the subject was in no wise inferior to the actual ideas entertained by the medical world at this day. A fact very worthy of remark is that Aspasia used to teach and recommend, in cases of inability to bear children up to the time of pregnancy, on account of malformation of the generative system, such means as modern writers would never dream of. But I shall not detail them, but merely refer the curious to

"*Actii medici Graeci contractæ ex veteribus Medicinæ Tetrabiblos per J. Cornarium Lugduni*, 1549, pp. 961–2–3. Sermo xvi., Caput xvi., et sequentia," and after a few more words concerning the history of the induction of abortion, pass to the more practical portion of our subject.

The induction of abortion, considered as one of the scientific resources of our art, fell into disuse and was rejected in times posterior to Aspasia, it not being spoken of by authors of the middle ages. We do not find it advised in any work posterior to that of Ætius, and it had probably fallen into total oblivion, when in the course of the last century Wm. Cooper, at the close of the relation of a case of Cæsarian section, which had proved fatal to the mother, submitted the following question to Dr. Hunter, to whom his paper was addressed:

"Before I conclude," says Mr. Cooper, "allow me to propose the following question: In such cases where it is certainly known that a mature child cannot possibly be delivered in the ordinary way alive, would it not be consistent with reason and conscience for the preservation of the mother, as soon as it conveniently can be done by artificial means, to attempt to produce abortion?"—*Med. Obs. and Inquiries*, vol. iv., p. 271.

This new scientific principle, elicited by Mr. Cooper, was broached at a moment when the attention of the medical world was wholly occupied by a prior question of the same nature; viz: *the induction of premature labor*, which had already been approved of and sanctioned in a consultation of the most eminent men of the time, held at London in 1759, for the purpose of considering the moral rectitude, and the advantages which might be expected from this practice.

"Induction of premature labor" being applicable to a greater number of cases than "induced abortion," certainly offered more interest as regarded its results, and this explains why the former had been resorted to a certain number of times, and had already given rise to much discussion, and to numerous interesting publications, while the latter remained stationary—a mere proposition laid before the inattentive medical world.

On the other hand, there were a few individuals who, considering the disastrous results attending the Cæsarian section when performed in England, and seeing no chance for the "induction of premature labor" in certain cases of extremely contracted pelvis to save the child's life, and very little probability of its preserving the existence of the mother, on account of the preposterous and mangled operation she was obliged to undergo, seriously turned their minds to the adoption, and their skill to the practice, of the principle suggested by Mr. Cooper. Among these men John Barlow stands the most prominent—(see his letter in Medical Facts and Observations, vol. viii., pp. 187 and 192. December 16th, 1779.)

In Germany the process was accepted and rejected by practitioners equally celebrated—Paul Scheel, Mende, 1802; J. P. Weidmann, Carl Wenzel, 1818, etc., etc.

In France, the proposal of this new operation was, at first, the particular object of examination and free discussion. What could we expect to find in favor of "induced abortion" in a country where Baudelocque and his school had severely condemned, as immoral, the induction of premature labor, which they very unscientifically styled "an abortion?"—(see Capuron's work.) In fact, the attention of the French school was so completely engaged with the refutation of the principles on which the induction of premature labor was founded, that the proposition of *induced abortion* was left so completely in the shade that one is tempted to believe, that it escaped entirely unknown.

Notwithstanding the opposition of Baudelocque and of Capuron and their school, we see Foderé in 1813, Marc in 1821, and Velpeau in 1829, declare in favor of the justifiableness and the morality of induced abortion in certain instances.

The question might still be considered as having been an open one till Prof. Dubois proved to his colleagues of the French

Faculty, and to the world, even in a more satisfactory and conclusive manner than had been proved by Denman in the question of premature labor, the moral rectitude and the legality of the principle on which was based induced abortion in cases of contracted pelvis.

Before relating the case of induced abortion because of malformation, that we have lately witnessed at the clinique, I believe I shall not be performing a thankless task if I quote some of the very valuable remarks contained in Professor Dubois' memoir on this subject to which he referred when lecturing on this delicate point a few days ago. The memoir was published in the Gazette Médicale in 1843.

"Those who oppose artificial abortion have alleged that amongst the women in whom abortion is induced by any kind of violence, there are few who are saved or who do not run the greatest peril; that when they do not die from the primary accidents, they generally fall victims to consecutive accidents of a still more serious nature; that the resource of the Cæsarian section, by which one woman out of three, on an average, has been saved, is by far preferable to that of induced abortion; that by no means whatever can the accoucheur possess over the fœtus an absolute right of life or death; and least of all, that to cause the death of the fœtus in utero or to expel it therefrom by any means before the period at which nature endows it with the power to live its own life, is nothing less than a crime punishable by the laws.

"On the other hand, the partisans of the contrary opinion have answered that under certain very serious circumstances, when it would be proved that the mother and the child could not undergo without absolute danger, the process of parturition at full term, it is by no means immoral to bring on abortion, and between two unavoidable evils to choose the least; that in so serious an alternative, the imperfect or puny organization and existence of a fœtus scarcely endowed with any physical sensibility, enjoying no moral faculty, unconnected as yet with the world by any external tie, cannot be compared with the existence of the mother whose faculties are developed, who is bound to society by numerous ties, and whose preservation, for all these reasons, is assuredly the most preciously to be taken into consideration.—(*Foderé*, *Med. Legal*, vii., p. 63).

Professor Dubois assumed the ground that the arguments here brought forward by both parties were not of a nature to elucidate and far less to solve the question at hand. Among those contrary to the principle, he would show that the one declaring it to be inconsistent with our moral duties, was based on an erroneous, or rather exaggerated conception of the law, and that among the reasons urged in favor of the adoption of "induced abortion," some had appeared to him rather of a sentimental than rational nature, and therefore, although they merited his personal approbation, he thought they possessed no positive value in a discusion of this kind.

Professor Dubois then proceeded to examine what had been termed the illegality and the criminality of "induced abortion." He said that although he was very far from being desirous of diminishing in any way the scrupulous sentiments inspired in the Profession by a rigorous respect and regard for the laws, he felt it his duty to place the question on ground where it might be free from all those moral considerations, which in the minds of many would render the scientific discussion of the principle very difficult to enter upon at the present momemt.

Professor Dubois then remarked that abortion as it is understood, pursued and punished by the laws, is a secret art, criminal to the very mind of him who brings it on, as it is to the conscience of the woman who solicits or suffers the operation to be performed. Abortion induced by scientific means, on the contrary, is accomplished openly (*au grand jour*) with the intention of saving one of the two lives brought into jeopardy—an act which ought not to be offensive to the conscience either of him who induces abortion, in those cases, or of the unfortunate creature who consents to undergo the operation. And then, again, abortion is not the only process of our art that needs to be legitimatised by the intention of the operation. The wounds, the mutilations inflicted daily by the hand of the surgeon, and which are sometimes resorted to for the purpose of removing a suffering that might have been borne, and put health, nay sometimes life itself, in very great danger, would they not be considered criminal if they were inflicted by other hands but ours, if their execution originated in a guilty motive? However, the law has made no distinction in these cases—it is sufficient that the legislator should have placed the one under the denomination of *crimes*, and that the other should be rationally made distinct from them. Moreover, among the mutilations effected for surgical purposes, there is one nominally foreseen and very severely punished by the law—that is castration. Well, no distinction was made by the legislature, and it was, no doubt, deemed sufficient to indicate criminal castration, in order that all other kinds of castration, scientifically performed, might be the exception to the law.

Having thus dwelt at some length on the origin and progress of the principle on which induced abortion is founded, let us now turn to a more practical theme, and endeavor to elucidate the somewhat delicate question, in what cases ought this operation to be performed.

Leaving out of the present question the consideration of *tumors*, that may obstruct the pelvis and may be displaced, punctured, or removed with more or less facility; leaving out of our investigation those *diseases* of pregnant females (diseases of the circulatory system, the abdominal tumors, dropsy of the amnios, etc.,) for which cases the induction of premature labor may be deemed necessary and against which induced abortion would prove a useless remedy—for the reason that at an early period of pregnancy the above mentioned diseases have not generally acquired a dangerous nature,—we must limit our attention to cases of *contracted pelvis*, (similar to that actually under our observation at the

Hôpital des Cliniques, and lay down the conditions under which, the mother's life being incompatible with that of her offspring, we feel ourselves under the dire obligation of attempting to save her life at the expense of that of the fœtus. Such cases are not numerous, and may be defined as follows:

Cases in which the pelvis is so far contracted as to leave no possibility of extricating the fœtus per vias naturales, even by diminishing the size of its head, and where no other means remain for delivering the woman of a living child but the Cæsarian section.

The above condition exists when the smallest diameter of the pelvis is under two and a half inches, (French measure.) From two and a half inches down to two inches, the extraction of the fœtus can certainly be effected by embryotomy; but if we come to consider the cases of this nature, we shall be easily convinced that the extraction of a dead fœtus under these conditions is no less dangerous to the mother, than the Cæsarian section itself. Symphysiotomy would endanger the life of mother and child to about the same degree. Artificial premature labor would not be followed by a better result, the only advantage of the latter operation being in such cases, that the division of an incompletely developed fœtus would be somewhat easier to effect, and its extraction less injurious to the mother.

Now that we have laid down the rule as to the period of pregnancy at which it is best to act, the mother being in the condition to which we have alluded—and under what degree of contraction of the pelvis we are called upon to induce abortion, we shall, instead of reviewing the various methods used for this purpose, which I need scarcely remark are the same as those consulted in cases of induced premature labor, proceed to the case of the dwarf actually under the care of Professor Dubois, at the Hôpital des Cliniques, and learn from this most valued authority the reasons that influenced his choice of induced abortion, and the several means by which he endeavored to effect his purpose.

Case.—Julie Gros, ætat. 30 years,—laundress. This woman measures only three feet one inch in height. Great distortion of the lower extremities; articulations knotty.—The trunk is short; head large; chin very prominent; complexion dark; expression very intelligent. Her general health has always been good, with the exception of some intestinal irritation three years back.

In September, 1846, she came to the Hôpital des Cliniques pregnant for the first time. Dr. Cazeaux who, at that time attended the wards in Professor Dubois' absence, learned from the patient that she had menstruated for the last time in June preceding, and after having ascertained the condition of the pelvis, he resolved to induce abortion. Dilatation of the os uteri was effected by means of prepared sponge (that is a conical piece of sponge tied up very tight and destined to increase in bulk by being in contact with the mucous membrane of the cervix.) After this and also the administration of ergot of rye, the membranes were ruptured on the 15th October, 1846. Abortion then took place without difficulty, at about four and a half months. The patient rapidly recovered, and left the hospital on the 11th day, proffering her oath that she would never be again found in the same predicament. Notwithstanding this, however, in November, 1847, Julie Gros returned to the same ward, pregnant for the second time. Again she came to solicit of Professor Dubois his intervention to effect her delivery, under circumstances very similar to those of the year previous. Her menses had not been seen since the 20th of September. Special reasons made the woman believe she had become pregnant on the 20th of September. Very soon after that date she had remarked certain sympathetic symptoms of pregnancy.

On the 2d of December, Professor Dubois pronounced her to be gone with child about two months and twelve days. He remarked that the determination he should take must depend upon the state of the pelvis. The conjugate diameter was measured by the finger; it was found to be two inches and one line in extent. The conjugate diameter being so short, Professor Dubois examined the resources at his disposal, which in the actual state of things were as follows:

1st. Induction of abortion up to the seventh month included.

2d. Induction of premature labor.

3d. Operation performed with the view of enlarging the pelvic diameter. *a.* Pelviotomy. *b.* Symphysiotomy.

4th. Mutilation of the fœtus, or embryotomy.

5th. Cæsarian operation.

Then followed the discussion concerning the operation to be performed. Some of these processes, said Professor Dubois, must be excluded; for instance, induction of premature labor. For it to prove completely effectual, it would be necessary to have a viable child, and moreover that the child be able to pass through a pelvis, the conjugate diameter of which measured twenty-nine lines. The thing was impossible. Premature labor induced in this case, would be equivalent to induced abortion. In other words, we should have the trouble of inducing premature labor without being rewarded by the ordinary beneficial results which characterize that operation.

Was pelviotomy to be resorted to? or either of its two modes of execution, pubiotomy or symphysiotomy? We shall simply remark that in such contracted pelves neither one nor the other operation could sufficiently enlarge the cavity for the reception and passage of a fœtal head without doing great injury to the woman. The rupture of the sacro-sciatic ligaments would certainly follow symphysiotomy. Pubiotomy was still more out of the question with regard to its practicability.

Mutilation of the fœtus? At the term at which this pregnancy had arrived, Professor Dubois said that it would be a dangerous operation for the mother, and moreover, it would be a difficult undertaking for the accoucheur.

The choice therefore rested between two operations—the Cæsarian section and induced abortion. Let us, said Professor Dubois, make the discussion bear on this point; let us weigh well the reasons for and against the chances offered by each of these operations, bearing in mind the while, the personal benefit and social importance connected with the question.

The advantages of the Cæsarian operation are the following: No particular structure being opposed to its performance in this case; its being, according to statistics, successful in one case out of three.

Now, on the other hand, continued Professor Dubois, as to statistics, the cases reported are generally the fortunate ones; all cases of this kind having been published, while it is well known that most of the unlucky cases have never been made public. As an instance of what he advances the Professor remarked, that the seven unsuccessful cases that had occurred in his practice had not all been published. And then, again, it is a fact that the results of the Cæsarian operation differ widely, according to the countries in which it is performed. Thus it happens that to our knowledge there is only one successful case out of all those practiced in England. If we turn to France, where the operation has been more frequently performed than elsewhere, we find that at Paris, not one of Professor Dubois' operations has resulted successfully to the mother. We may therefore infer from this circumstance, that not a single mother is likely to escape in this city. As for the children, those who survived for a certain time or continued to live, owe it almost entirely to the excellent care taken of them—the others, abandoned and neglected, have been lost sight of, and no doubt have run all the risks incident to their condition, which by the fact of their premature birth are rendered far more serious than those of children coming into the world by the process of nature. In conclusion, we may say that the Cæsarian section when performed in Paris is fatal to the mother, and that the children who in cases of this kind are often abandoned very soon die.

In the present case the woman says she would not be able to keep her child. As to bringing up the child himself, Professor Dubois said that he had already reared two individuals whom he had ushered into the world by the Cæsarian section, one of whom was a fine tall soldier at this time, but that as to adopting a third child, it was an undertaking of rather too delicate a nature to be immediately decided upon.

Finally, there remains abortion. Here Professor Dubois dwelt at some length on the subject of the first part of this paper; the high importance of the question, the moral rectitude of the principle we have adopted according to scientific rules, etc.

Speaking of the various processes hitherto employed to effect this end—abortion—he remarked that the criminal one consisted in the administration of substances acting on the uterus by determining congestion in that organ, or else in operative processes, such as perforation of the ovum.

His opinion was that one of the best, as well as the most scientific methods, was the one resorted to in cases where it was wished to induce premature labor—that is the dilatation of the cervix by prepared sponge, (Kluges' method.) This method he esteemed much preferable to that acting on the principle of rendering the uterine system congested, as by warm baths, ergot of rye, etc., etc. If the former plan was not effectual, the old method then presented itself; namely, the perforation of the membranes. All these procedures tended to one and the same physiological end: to wit, the inducing of uterine contraction, whether we choose by the last process to evacuate *all* the amniotic fluid, as Clarke used to do, or only a small quantity of the waters as Meissner says he does now, by means of a curved sound conducting a trochar. To either of these two procedures Professor Dubois would prefer employing an intermediate one—Hamilton's method—which consists in the introduction of the finger into the os uteri and the detaching of the membranes from their connexions in the neighborhood of the orifice. This method has of late been somewhat modified by Professor Simpson, of Edinburg, who uses a curved sound of large calibre to effect the detachment of the membranes from the corresponding uterine surfaces. Before, however, having recourse to either of these methods, Professor Dubois determined to try the effect of galvanic shocks on the uterus, with the view of producing primary contraction of that organ, or its secondary action, after having killed the fœtus by the shocks, and its acting as a foreign body *in utero*.

The apparatus used for this purpose was an electro-magnetic machine. The conductors being placed alternately on each side of the uterus through the abdominal parietes; again one on the fundus and the other on the cervix, *per vaginam*, the shocks were gradually increased, during one or two minutes, till they became insupportable. The experiment was renewed twice—the third time the patient was very refractory, and refused to inhale chloroform to allay the pain, when Prof. Dubois concluded to postpone the further trial of the galvanism for a fortnight, in order to ascertain if the uterus still continued increasing; finding that it did so, and being unwilling to inflict any more suffering upon the patient, who still continued exceedingly intractable, he entirely renounced this method, which he was the first to apply with a view of inducing abortion, and which he believed failed to produce its desired effects in this instance because of the want of power in the instrument used, and the indocility of the patient. On the 8th of January, 1848, he proceeded to accomplish his purpose by separating the membranes from the lower part of the uterus, which he effected by the introduction and gentle rotation ,within the cavity of the uterus, of a curved sound of large calibre, avoiding, as much as possible, the rupture of the amniotic bag, which he hoped would be very effectual in aiding the dilating effect of uterine action.

Professor Dubois operated in presence of the class at 9 o'clock, A.M. A small quantity of blood was lost during the operation, and gave rise to a belief on the part of the Professor, which the inspection of the placenta afterwards proved to be well grounded, that this body had been lacerated by the sound. The membranes, however, were not ruptured. During the day, uterine contractions began to be felt at long intervals. In the course of the following night, and particularly in the morning of the 9th, the pains set in pretty regularly and strong. They continued so during the day, and at 5 o'clock, P.M., dilatation being very nearly complete, the bag of waters was opened; delivery took place spontaneously between 5 and 6 o'clock, about thirty hours after the operation.

In his clinical lecture of the 11th January, Professor Dubois exhibited the placenta, a portion of which appeared lacerated, owing, no doubt, to the action of the sound which had been employed. He remarked that the labor had lasted a longer time than in the average number of labors, but in this case, labor was not effectually established before 5 o'clock on the morning of the 9th, the true pains having lasted, therefore, only about twelve hours, the time generally allotted to natural labor. In this preternatural case, said Professor Dubois, the first pains had been employed in preparing the cervix to dilate, as it had been surprised in a state quite unmodified, with regard to the process which was about to take place, and in which it had so important a part to perform. The fœtus came by the vertex, and pushing the placenta before it—another proof that the placenta had been detached during the artificial destruction of the uterine connexions, and had made its way to the orifice before the fœtus.

The patient has fared pretty well. Some abdominal pains and fever were present the second day after confinement. Two applications of ten leeches each were sufficient to restore a healthy condition. The sympathetic phenomena in the breasts which were noticed to be attempted, were more than realized on the third day, but very soon subsided. Things have continued to go on well, and the patient was expected to leave the hospital this morning, January 21st.

Few of the physicians of Paris appear to me more interested in the cure of diseases than M. Trousseau, and there is not among them one whose clinical lectures afford more wholesome and valuable therapeutical instruction. In one of his late lectures at the Hôpital Neckar, among other things, he made some remarks on *erysipelas in new-born children*, which I propose to report.

Erysipelas in new-born children, the Professor remarked, is a disease of frightful gravity; it attacks all classes indiscriminately, the rich and the poor, and it is almost invariably mortal, whether the children be in the condition of the most prosperous health, or whether they be cachectic. I have seen only three infants recover during the last nine years; the one was five months, the second three months, and the last a year old. All save these have died.

Although it is our object, this morning, to call your attention especially to erysipelas in infants, we may state to you certain divisions depending on the age of the patient, which experience has taught us to regard as being about as generally correct as general laws usually are: In the infant, then, (from one to two months of age) erysipelas is excessively grave; later it offers less gravity; and finally, towards adolescence, it does not differ much from that of the adult. You see a child who is not sick; the mother begs you, however, to examine it. She has seen in dressing it something not altogether natural about its genital organs, and she wishes you to look at it; you do so, and find, if it be a boy, the penis a little swollen and red; if it be a girl, the vulva slightly tumefied; detecting but this, you do not suspect its gravity, and hence do not distrust yourself. At your visit the following day, you are surprised to find the parts tumefied, the penis, the scrotum, or the vulva, swollen; there is no fever, no loss of appetite; you believe it an insignificant affair, and order a cataplasm. The next day the swelling extends a little towards the umbilicus, the thighs, the pelvis; this begins to be unpleasant; the infant now cries a little, sleeps a little less than usual, but does not vomit; it sucks, has no fever, no cough, and no cerebral phenomena. When you return to your little patient, you are struck with its discoloration and pallor; it cries, it has not slept, it sucks with avidity, because it is thirsty; but there is neither diarrhea, nor vomiting; the swelling gains the thighs and the legs. The next day the paleness is greater; the infant no longer sleeps, and does nothing but cry; the erysipelas reaches the trunk, the feet, attains the neck, spreads itself, and it is rarely that death does not supervene the sixth or seventh day.

I have spoken to you of this open and undisguised march of the affection, in order to strike your minds by a description which shall cause you to mistrust yourselves, and prevent your feeling a deceitful security while the child is dying.

The prognosis is always very grave, and the reason is that the erysipelas is often accompanied by a peritonitis which sometimes resembles the puerperal species; it does not supervene immediately in the infant, but comes to cover the erysipelas; arises at the end of four or five days, and kills in twenty-four hours. It is a serious, indeed, an invariably mortal disease.

Furthermore, there very often exists an umbilical phlebitis; the umbilical vein becomes inflamed, and the veins of the liver become filled with pus. The infant is thus placed in the most alarming conditions that any one attacked with erysipelas can be; in fact, if in individuals laboring under deep-seated abscesses, gangrene, phlebitis, etc., an erysipelas supervenes, you announce death, and the patient dies. In the child, something analogous to this occurs; the umbilical cord becomes detached as a gangrened portion; it cannot be otherwise detached; then is there a noncicatrized umbilical wound, and the erysipelas almost always commences at this point. Under ordinary circumstances, this condition of wound is nothing; but at the moment when puerperal fever reigns in the hospital there is a connection between the disease of the mother, who dies of puerperal fever, and the disease of the child who dies of erysipelas.

A woman, seven months gone, occupying one of the beds of our ward, has had, for fifteen days past, incessant vomitings, her stomach being incapable of retaining anything whatever. We will seize this occasion of saying something of the vomitings which supervene in the course of pregnancy. I should remark, however, that in the patient to which I have just made allusion, there has existed a diarrhea for four or five months, and that she offers something which does not resemble the vomitings of which I propose speaking.

It is very common to see women attacked, during pregnancy, with vomitings which nothing is capable of arresting. We saw one instance last summer, and if the cases were collected we believe there would be many similar to it, of a woman who finally died under these circumstances from inanition.

This grave disease has been combatted by very various means and medicines, especially by alcoholics. And there is no doubt that very strong alcoholic drinks suffice, in certain women, to arrest the vomiting. The experiment has been made to ascertain their *modus operandi*, but I am unable to tell you by what mechanism they act. This medication, however, like the others, is often inefficacious; the disease progresses, and the women perish or abort.

Five years ago, a lady, pregnant for the first time, who for six weeks had vomited both liquids and solids, called in M. Bretonneau. He found the patient in a most alarming state; the affection progressed very rapidly, and threatened to become inevitably fatal. An accident put M. Bretonneau upon the right track. This woman when questioned complained of sharp uterine pains. In a primipara, the fibres of the uterus are not broken in, if you will allow the expression, are not habituated to the process, and allow themselves to be distended with difficulty; and it is this which causes the pain. M. Bertonneau thought that the uterine pains were the cause of the other symptoms, and that if he succeeded in mastering them, he would overcome the sympathetic vomitings of the patient. Acting upon this idea, he covered the hypogastrium repeatedly with a mixture of belladonna; the vomitings ceased the same day. and recovery ensued. Sometime afterwards, he had occasion to observe another case where the pains of the uterus did not exist; but he thought that even if the brain did not perceive the pains of the uterus, the ganglia might take note of them and reaction occur. To modify these accidents he believed it sufficient to prescribe the belladonna mixture, and was again gratified with complete success. The result of these and similar cases justifies him, he thinks, in laying down the following principle:

Whenever in a woman, pregnant for the first time or many times, vomitings supervene during the course of gestation, frictions should be made upon the hypogastrium with a mixture of belladonna, and the vomitings will cease.

About a year and a half ago Dr. Ems called me in consultation in the neighborhood of Versailles, and after we had examined a patient laboring under rheumatism, he spoke to me of a woman who gave him great uneasiness, and who appeared to be going to die of hunger. This woman, he said, was in the seventh month of her pregnancy; her stomach was totally unable to bear anything, and she was in what seemed a hopeless state. I told him of the treatment of Bretonneau in such cases; he tried it; the next day the vomiting ceased; the patient was able to take aliment, reached the full term of her pregnancy, and gave birth to a child. It is now six months since Dr. Ems related to me this fact, at which time he cited me another patient in a similar condition, whom he had also treated with success by the same remedy. Dr. Duclos has seen two women attacked with obstinate vomitings, in whose case all medication had failed till he used the belladonna, which, true to its end, cured them.

In what manner does the belladonna act? I confess it is impossible to determine. Can it be supposed that the fœtus in being developed, painfully distends the fibres of the uterus; that the vomitings are sympathetic, like those which supervene in cystitis, for example? This is possible. Whether it be this or something else, it is upon this hypothesis that M. Bretonneau has employed his remedy. He has promulgated his theory, and has endeavored to confirm it by facts. The fœtus distends the uterus; the nervous ganglia take cognizance of it, and sympathetic vomitings are the consequence. This is his theory, which you may adopt or not, but which you must all admit conforms with marvellous exactness to the therapeutical results.

The case which occupies us is neither so clear nor so well marked as the others. The patient has had diarrhea for four months, a sensation of a hot iron and scaldings in the stomach, vomitings, but she had none of these in a preceding pregnancy; it is important not to confound the vomitings which supervene in gastro-enteritis with those which are sympathetic with the uterus. That the vomitings in the case before us are sympathetic is possible, but I do not venture to affirm that it is so.

The remedy of such seeming efficacy in the cases described, will possibly fail here. I am going to assume that there is a gastro-intestinal affection—to suppose that there is excess of development of acid in the stomach and the superior portion of the small intestine, and for this employ magnesia. This substance in contact with it saturates the acid, and modifies the organ in such a way that it secretes less, and the burnings of the stomach disappear. If the gastro-enteritis disappears and the vomitings persist, I will suspend the magnesia, concluding that the vomitings are sympathetic, and will proceed to the confirmation of my theory by the method that I have pointed out to you. It is of great importance to discriminate clearly and correctly, as you see, for the medication may appear bad, while it has only been improperly employed.

*Rheumatism Cured by Quinine.*—A woman, five weeks after delivery, entered the wards of M. Trousseau a short time ago, laboring under an attack of acute articular rheumatism, which had commenced five days before. The heart was not involved, there being no symptoms of either endocarditis or pericarditis, though to a very violent fever there was joined hydrarthrosis of both knees, and swelling of the sheaths of the tendons of the feet.

Without any previous medication having been employed, sulphate of quinine, administered from the second day of the entry of the patient into the hospital, procured, in the space of four days, a marked amelioration in her condition. Incapable of doing so before, she was able to walk on the fifth day, at which time the fever had disappeared, and scarcely any fluctuation could be felt in the articulations.

M. Trousseau remarked: Here, now, is a rheumatism which has been cured in a very few days. But it is by no means the case that things usually pass thus; most often the symptoms persist during three weeks, more or less. When the rheumatism improves you must pursue the same therapeutical course as in intermittent fever; if you immediately stop the administration of the quinine there will be a relapse, and you must never forget that relapses often last longer and are more rebellious than the original attack. This return of the affection is to be prevented by the administration of quinine in the same doses, at intervals of two, three, and four days, and continued after the subsidence of the pains in a preventive manner.

You should act upon the idea that rheumatism is not a local affection, and that an individual is not cured when the pains have completely disappeared. We see the buffy coat persist so long a time, that we are obliged to employ medication against the rheumatism which would still appear, and it is in this point of view that the continuance of the quinine is important in such cases.

So long as the patient has sweats and his appetite does not return, and there is something wrong in the pulse, you must treat the diathesis. When we employ venesection, we do not treat the rheumatism; we occupy ourselves with the diathesis, in virtue of which the liquids and the solids take on rheumatism. When we make use of quinine we attack the whole economy—and that particular condition of it called diathesis, out of which the pains and other phenomena in the articulations arise. The diathesis may persist two or three years after all manifestations have ceased, and we are obliged to act against it in the same manner that we act against the syphilitic, scrofulous, and other diatheses when the other manifestations have disappeared.

The same rule is applicable to the miasmatic febrile diathesis; that is to say, to that diathesis in virtue of which an individual possesses within himself the conditions necessary for the production of intermittent fever. A man inhabits a marshy country and quits it without ever having had intermittent fever—he settles in a district where the fever has never existed, and three or four months after he does so he is attacked with intermittent fever. Why is this? Because he preserves the the febrile diathesis. At the end of a certain time this individual is relieved of his paroxysms by the sulphate of quinine, but is he cured of the diathesis? No. If you suddenly cease the quinine even when the paroxysms have not been seen for eight days, the fever returns; from whence you see that the disposition by virtue of which the individual had intermittent fever, the diathesis, still exists. It may be most correctly compared to the rheumatismal diathesis, and if it be the rule in the one case to insist on the employment of quinine during three months or more, you must comprehend that the same is required in the other, whether it be a little more acute or a little more chronic.

*Chloroform.*—After my long letters on Ether, I deem it useless to say much of Chloroform, as an agent to allay pain in surgical operations. Suffice it then, to say, that chloroform has completely supplanted ether in the Paris hospitals; that the surgeons are unanimous in according to it a decided superiority over this substance—a superiority consisting, briefly, 1. in its more agreeable taste and odor; 2. in its being less irritating than the ether to the air passages; 3. in its desired effects being more speedily obtained, more complete, continuing for a greater length of time, and passing away when they once begin to do so more readily than those of ether; 4. and lastly, its more simple mode of administration, no apparatus appearing to succeed so well as the simple concave piece of sponge.

*Eschars of the Sacrum.*—From the time that M. Blandin was a student of medicine, he says he was struck with the sudden manner in which eschars of the sacrum often produce death, and the important fact was referred to in his Surgical Anatomy twenty-two years ago. In a late clinical lecture he remarked, that he believed he had found an explanation of the fact in the following circumstances:—the point which suffers most from pressure in the dorsal decubitus is that which corresponds to the union of the sacrum and coccyx, which is precisely the point where the vertebral canal is formed only by the posterior sacrococcygeal ligament. This disposition of things allows the sphacelus to reach with great ease the *cul-de-sac* of the arachnoid; from whence the cavity of this membrane is opened, and the air, pus, and sanious matter penetrate. From this there results a violent inflammation which produces the phenomena of paralysis in the rectum, bladder and inferior extremities.

While showing the gravity of eschars of the sacrum, M. Blandin urges the precautions by which they are to be avoided, consisting in unwearied care, cushions, etc. etc.

*Comparative advantages in Children of Lithotomy and Lithotrity.*—The opinion of M. Guersant, Jr., of the Hôpital des Enfans, concerning the comparative advantages of Lithotomy and Lithotrity in children, is summed up in one of his clinical lectures. After having successfully lithotritized a boy of seven years old, he remarked: The question here presents itself, whether one should prefer in children lithotomy or lithotrity? Some time ago I presented to the Academy of Medicine a comparative table of which the following is the substance: of 51 calculous children that I have seen here, I have lithotomised 37. Of this number 30 recovered and 7 died; but four of these died in consequence of an intercurrent disease. I have lithotritised 8; 5 of these have recovered and 3 died; but only one died on account of the operation. In the remaining six the calculi were seated in the canal of the urethra, and I had no difficulty in removing them.

You will remark that so far as my experience goes, the results have been equally fortunate in the two operations; nevertheless I must express my preference for lithotomy when the calculi are voluminous, and lithotrity when they are small. I know that lithotrity offers certain inconveniences; thus, for example, it sometimes happens that there remain after the operation fragments of considerable size, and that, the neck of the bladder being easily dilated and the emission of urine being performed with force by children, the fragments may become engaged in the canal, in such a manner as to be incapable of either advancing or retreating, and thus render a new operation necessary. But this, we think, does not happen sufficiently often to justify our relinquishing the aid to be derived from lithotrity.

*Prussian Blue in the Treatment of Neuralgias.*—Prussian blue, a double salt which has received the names of ferrocyanate, double cyanate of hydrate of iron, etc., has never been very greatly employed by physicians. It is, says M. Bouchardat, a very important product for dying, but inert in medicine. This author adds, that it was once extolled in the treatment of intermittent fevers, in doses of from one to ten grains; as also in chronic diarrhea, in doses of one or two scruples; and in epilepsy, in doses of from six to ten grains. The sway of this salt soon passed away, and has never since revived, owing partly, perhaps, to its having been over-praised. M. Rayer has, however, been experimenting with it lately, and, it appears, with a success which has induced him to continue and extend his trials. This celebrated physician does not employ it in the large doses I have mentioned, but confines himself to the administration of it, in the form of powder, in doses of from one grain to one and one-fifth of a grain, which he continues for one or two months. The affections in which he has exhibited it belong to the order of neuralgias—principally those of the face—and cephalalgias.

Although this medicament has not been used to a sufficient extent to warrant us in pronouncing with any confidence upon its complete efficacy, in diseases so rebellious as the neuralgias, its use appears to have been followed by some decidedly beneficial, without any unpleasant, results.

*Acupuncture in the Treatment of Blemishes of the Cornea.* A Spanish Medical Journal, the *El Regenerador*, mentions that a Dr. Perez has used acupuncture in the treatment of a number of cases of spots of the cornea, with great success. The following is the manner in which he proceeds: The patient being placed as for the operation of cataract, the surgeon fixes the eye, either by an instrument or by means of the finger of an assistant. He then takes the needle, and holding it like a writing pen—(sometimes he plunges the extremity in a solution of 12 drops of prussic acid to 3 scruples of distilled water) he introduces it at a very acute angle, of 2° to 4°, at each of the extremities of the diameters of the cornea, at half a line from the union of this membrane with the sclerotica. He carries it sometimes to the second order of lamella which constitute it; sometimes to the membrane of the aqueous humor, and sometimes to the crystalline body. The needle is always allowed to remain in its place during a space of time varying from two to five minutes.

After having withdrawn it, it remains to combat the accidents of reaction according to the degree of their intensity.

Paris, Jan. 27, 1848.

